# Substrate-Dependent Performance of ZnTTBPc–PMMA Composite Films on Rigid, Flexible, and Sustainable Materials for Wearable Devices

**DOI:** 10.3390/polym17111574

**Published:** 2025-06-05

**Authors:** María Elena Sánchez Vergara, Ismael Cosme, José Ramón Álvarez Bada

**Affiliations:** 1 Faculty of Engineering, Universidad Anahuac México, Av. Universidad Anáhuac 46, Col. Lomas Anáhuac, Huixquilucan 52786, Estado de México, Mexico; ramon.alvarez@anahuac.mx; 2 Polytechnic University of Cuautitlán Izcalli, Av. Lago de Guadalupe, Colonia Lomas de San Francisco Tepojaco, Cuautitlán Izcalli 54720, Estado de Mexico, Mexico; 3 National Institute of Astrophysics, Optics and Electronics (INAOE), Luis Enrique Erro #1, Tonantzintla 72840, Puebla, Mexico; ismaelcb@inaoep.mx

**Keywords:** semiconductor composite film, substrate, topography, optical properties, fluorescence

## Abstract

The purpose of this work is to evaluate the potential use of zinc 2,9,16,23-tetra-tert-butyl-29H,31H-phthalocyanine (ZnTTBPc) embedded in polymethyl methacrylate (PMMA) and deposited on different substrates in active films for wearable device (WD) applications. The inclusion of PMMA as a matrix facilitates the incorporation of ZnTTBPc. The composite films were deposited by drop casting on PET, glass, and n-type silicon, as well as on innovative substrates, such as palm leaves and polyester. Regarding the composite films, surface analysis using SEM and AFM revealed substrate-dependent differences in film roughness, grain distribution, and crack formation, highlighting the influence of substrate morphology and drying dynamics on the structural integrity of the composite films. With respect to fluorescent and optical behavior, the highest fluorescence intensity (2573) and reflectance (75%) were obtained for the film deposited on palm, while the lowest optical band gap (1.52 eV) was found in the film on polyester fabric. Substrate–film interactions and deposition dynamics play a critical role in determining the structural integrity and topography of composite films, which, in turn, influence optical properties, fluorescence, and band gap. The multifaceted properties of all tested systems with the film structure, substrate/ZnTTBPc–PMMA suggest new possibilities for wearable electronics applications.

## 1. Introduction

Biomedical instrumentation systems frequently operate with a device consisting of a measurement-facilitating energy source, a sensor that converts the measured signal into an electric signal [1], and a signal conditioner that amplifies and filters the signal for data deployment [2]. These biomedical instrumentation devices use sensors for sample information collection and further analysis [1]. Transducers are components that transform signals from one energy form into another [2,3], while sensors are specific types of transducers transforming physical signals of various types into electric signals [2,4]. Sensors must satisfy characteristics such as specificity, simplicity, durability, high sensitivity, and reduced size, as well as low cost. There are several classifications for sensors, depending on factors such as whether the delivered signal is analog or digital [2,5]; the property they are capable of measuring (such as temperature, pressure, proximity, acceleration, and other gas and chemical features, among others) [2,6]; their applications, which could also be related to industrial process control and automation [2,7] or non-industrial processes, as may be found in medical equipment [2]; and the requirements related to the active or passive delivery of energy or power [2,8].

Among the most frequently used sensors are those employed to measure physical properties such as capacitance, resistance, and electric conductivity, as well as temperature, position, force, weight, or light [9]. While their structures are quite different from each other, most sensors typically have two fundamental components, i.e., a casing and a detection medium [10]. The detection material, or medium, of the transducer is applied to detect changes in some property of the material and convert them into an adequate physical signal for later analysis [3]. Among the detection materials, one may find noble metals, metallic–oxide semiconductors, carbon nanomaterials, metallic dichalcogenides, graphitic carbon nitride, and complex compounds [11]. In the case of wearable devices (WDs), which have become increasingly popular during the last decade in the biomedical and health industries [12,13], the detection material may vary depending on the sensor type. Nevertheless, most WDs require flexibility and/or stretching capacity for adaptation to the human body [14,15,16,17]. WDs provide a noninvasive means to perform the constant monitoring of physiological signals in real time [14,15,18,19]. They can be carried in clothing or other body accessories, and through their sensors, they are capable of monitoring physiological and biochemical parameters [12,19]. Flexible electronics is frequently involved in WDs, as their components may require mechanical resistance, low weight, flexibility, portability, and an ergonomic character [16,17]. Flexible electronics devices generally involve components such as a substrate, an active layer, and an interface [17]. Due to the conductivity and flexibility that they provide, printed carbon nanomaterials, such as graphene [17], and liquid metals, like eutectic indium gallium (EGaIn) and Galinstan (Ga/In/Sn) [17], are widely used in wearable-type sensors as active-layer materials. On the other hand, mainly due to the significant toxicity and high production costs of these materials, organic semiconductors have become a viable alternative as constituent materials for the active layers in WDs [20,21].

**Figure 1:**
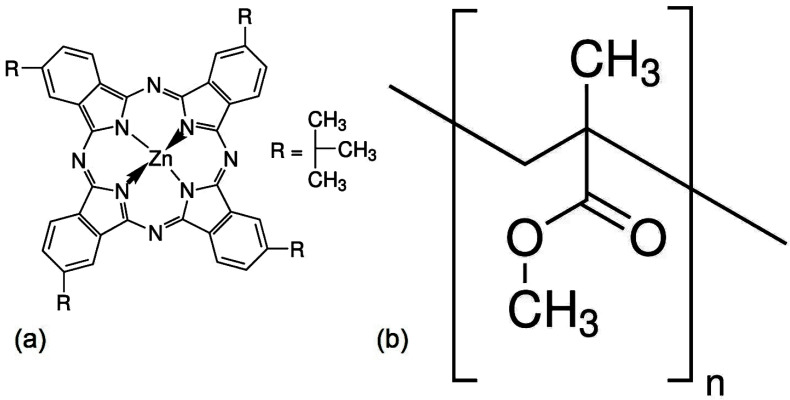
Molecular structure of (**a**) zinc 2,9,16,23-tetra-tert-butyl-29H,31H-phthalocyanine and (**b**) poly (methyl methacrylate).

Metallic phthalocyanines (MPcs) have specifically been used as detection layers due to their conducting properties [22]. MPcs are planar aromatic organic macrocycles that contain 18 delocalized π electrons [23,24]. The conjugated π system provides valuable optical and photoelectric properties [24]. This, together with their mechanical flexibility and their high thermal and chemical stability [24,25,26], makes them suitable for applications in optoelectronic devices such as WDs [24,25,26]. Given the diverse properties and applications of MPcs, special importance should be given to zinc phthalocyanines (ZnPc) and their derivatives, which are used as organic semiconductors with nonlinear optical properties such as high triplet quantum yield and light absorption coefficient [26]. In particular, the ZnPc variant known as zinc 2,9,16,23-tetra-tert-butyl-29H,31H-phthalocyanine (ZnTTBPc) (Figure 1a) contains four isoindole units joined by nitrogen atoms to the zinc aromatic ring [27,28,29], with tert-butyl groups in the extremes. These external radicals improve solubility in organic solvents [30,31,32], while the Zn core provides high thermal stability [30] and moisture resistance [30,32], allowing for their use in WDs. Furthermore, ZnTTBPc has an ionization potential of 1.8 eV, a low band gap of 1.5 eV [33], and important optical properties, such as a high optical absorption coefficient within the range of visible light [30,32,33]. It also has photoconductive properties, since light exposure increases its electric conductivity [30]. Due to these properties, as well as its low density and ease of deposition on flexible and rigid substrates, ZnTTBPc may be used in medical applications [31], optoelectronics [30,31], photovoltaics [32], and photodynamic therapy [34].

On the other hand, it seems to us that there has been limited use of ZnTTBPc in WD fabrication. For this reason, it is an objective of this work to evaluate its potential use as thin films that may be deposited over different substrates for WD applications. To increase the grip on the substrate of the active layers and improve their resistance under service conditions, the fabrication of composite films where ZnTTBPc is embedded in polymethyl methacrylate (PMMA) (Figure 1b) is proposed. We propose a PMMA matrix because it is lightweight and transparent, has good molecular ordering, and is compatible with flexible and rigid substrates. Moreover, its film-forming ability, optical properties, and biocompatibility with human tissue make it a suitable material for WDs. The contribution of this work can be found not only in the fabrication and characterization of ZnTTBPc–PMMA composite films but also in the type of substrate used. Substrates comprise a fundamental part in the building of WDs, so it is very important to manufacture thin-film systems in a wide variety of substrate geometries and materials [35]. The use of the following active substrate materials has been reported: (i) polymeric substrates that are mechanically flexible, lightweight, and conformable supports [35,36,37]; (ii) shape memory materials with the ability to change their shape upon the application of external temperature [38], magnetic field [39], and chemical conditions [40]; (iii) transient substrates with the ability to biodegrade and bioresorb for smart medical implants [41] while favoring a sustainable approach to electronic applications [35]; and (iv) textiles substrates, due to their ability to conform to the human body [42]. However, these substrates with ZnTTBPc films embedded in a polymeric matrix have been rarely used, and for this reason, conventional substrates (glass and silicon), a polymeric substrate (polyethylene terephthalate), a transient substrate (palm leaf), and fabric (polyester) are proposed in this study. Although there are different methods for preparing composite films of polymer matrix and semiconductor particles, such as spin coating, dip coating, and layer-by-layer assembly, to mention a few of the most important, in this study, we propose the manufacture of ZnTTBPc–PMMA films by drop casting. This is because, compared with previous methods, this is a low-cost, easy, single-step technique that can be used with the variety of substrates studied in this work [43].

## 2. Materials and Methods

**Figure 2:**
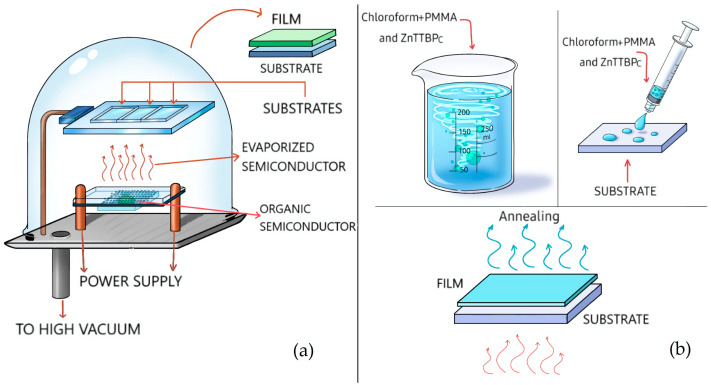
Schematics of the techniques (**a**) high-vacuum sublimation and (**b**) drop casting.

Zinc 2,9,16,23-tetra-tert-butyl-29H,31H-phthalocyanine (ZnTTBPc: C_48_H_48_N_8_Zn) and poly (methyl methacrylate) (PMMA; [CH_2_C(CH_3_)(CO_2_CH_3_)]_n_) were obtained from commercial sources (Sigma-Aldrich, Carlsbad, CA, USA). All the films prepared for this work were deposited on glass substrates, n-type silicon, polyethylene terephthalate (PET), polyester fabric, and palm leaves. Before being used for thin-film deposition, all substrates were cut into 2.5 × 3 cm pieces. Some substrates were subsequently subjected to surface preparation treatment: the glass substrate was washed sequentially in an ultrasonic bath of chloroform, isopropanol, and acetone. The silicon substrate was then washed with a “p” solution (15 mL of HNO_3_ and 10 m of HF in 300 mL of H_2_O) and acetone, and the PET substrate was stripped of its protective layer. Finally, the palm was sanded and blasted to obtain a streak-free surface. Different types of films were prepared: pristine ZnTTBPc films, pristine PMMA films, and ZnTTBPc–PMMA composite films. The pristine ZnTTBPc thin films were deposited by high-vacuum sublimation (Figure 2a), which was performed within a high-vacuum chamber at 10^−5^ torr, with a system involving mechanical and turbomolecular pumps and a microbalance (Intercovamex, S.A. de C.V., Cuernavaca, Morelos, Mexico). This system was connected to a thickness gauge. ZnTTBPc was introduced into the chamber in a tungsten crucible, heated until there was a phase change from solid to gas, and finally deposited on the substrates at a deposition rate of 0.7 Å/s. Pristine PMMA was obtained from a dissolution of 1 g of polymer in 10 mL of chloroform. The solution was added by using the drop-casting technique (Figure 2b) on each substrate surface; subsequently, the chloroform was removed by annealing at 50 °C for 5 min. The ZnTTBPc–PMMA films were obtained from a dispersion of 0.5 g of ZnTTBPc in a solution of 10 mL of chloroform with 1 g of PMMA. After agitating the ZnTTBPc–PMMA dispersion, 6 mL was applied by the drop-casting technique on all the substrate surfaces (see Figure 2b). Immediately afterwards, the films were annealed at a 50 °C temperature for 5 min. Film thickness was measured by stylus profilometry using a Dektak model XT (Bruker Corporation, Boston, MA, USA) on the glass substrate, where a mechanical step could be created due to its rigid and smooth surface. The resulting average thickness was approximately 3 µm. For the IR-spectroscopy characterization, a Nicolet iS5-FT (Thermo Fisher Scientific Inc., Waltham, MA, USA) spectrophotometer was used. Absorbance, transmittance, reflectance, and the Kubelka–Munk function were evaluated with a 300 Unicam ultraviolet–visible (UV–Vis) spectrophotometer (Thermo Fisher Scientific Inc., Waltham, MA, EUA). The topographic and morphologic features of the films were analyzed by using atomic force microscopy (AFM) on an NTEGRA platform (NT-MDT Inc., Liestal, Switzerland), as well as a Hitachi SU3500 (Hitachi, Tokyo, Japan) scanning electron microscope (SEM). Film fluorescence was measured with an FP-8550 spectrofluorometer (Jasco International, Tokyo, Japan).

## 3. Results and Discussion

### 3.1. Structural and Optical Characterization of Pristine ZnTTBPc Film and Composite ZnTTBPc–PMMA Film

**Figure 3:**
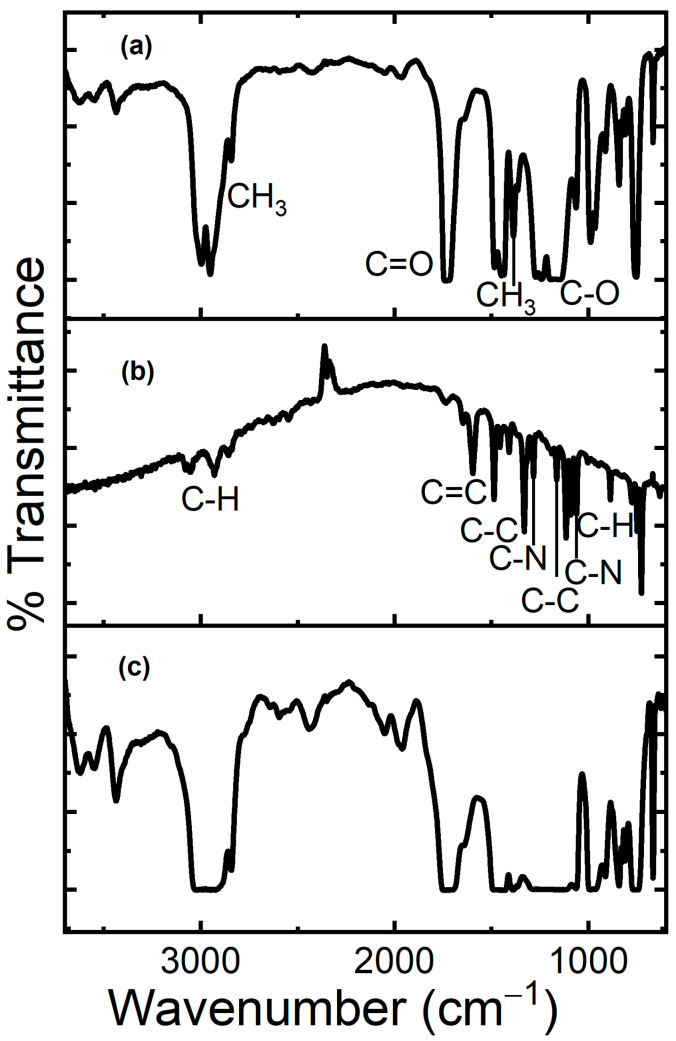
IR spectra of (**a**) pristine PMMA, (**b**) pristine ZnTTBPc, and (**c**) ZnTTBPc–PMMA films.

Upon the deposition of the pristine ZnTTBPc and ZnTTBPc–PMMA films, IR spectroscopy was carried out to determine whether any of the components was degraded. Given that the deposition conditions of the two types of films were the same for the different substrates, Figure 3 only shows the IR spectra of the films over n-type Si. Figure 3a shows the PMMA film spectrum as a reference, where the bands at 2950 and 2843 cm^−1^, related to the stretching vibration frequency of CH_3_, can be found; the band at 1385 cm^−1^ is related to the bending vibration frequency of CH_3_ [44]. The band at 1727 cm^−1^ corresponds to the tensile vibration frequency of C=O, and the band at 1155 cm^−1^ represents the tensile vibration frequency of C-O [45]. Flat bands at 1727 cm^−1^ and between 1280 and 1140 cm^−1^ can be found in the spectrum due to several reasons, such as the presence of carbonyl in the polymer structure and the vibrations of this polymer that may overlap. Additionally, PMMA has an amorphous structure, which may widen the bands because of variations in the intermolecular interactions [46,47]. On the other hand, the pristine ZnTTBPc spectrum shown in Figure 3b features the band at 3052 cm^−1^ related to the asymmetrical stretching of the aromatic C–H bond, as well as the band at 1587 cm^−1^ related to the vibration due to C=C benzene bond stretching, the band at 1409 cm^−1^ due to the isoindole stretching vibration, the band at 1331 cm^−1^ due to the C-C planar vibration of the aromatic ring, the 1284 cm^−1^ band due to the C-N planar stretching vibration, the band at 1167 cm^−1^ due to the C-C planar isoindole bending, the band at 1114 cm^−1^ due to the C-H out-of-plane bending in the aromatic rings, the band at 1059 cm^−1^ due to the C-N planar bending of the imine isoindolic units, and the band at 887 cm^−1^ due to the C–H planar bond bending of the aromatic ring [48,49,50]. The vibrations in the region below 800 cm^−1^ are sensitive to small differences in the short-range interactions between adjacent ZnTTBPc molecules, which permits the identification of the 725 cm^−1^ band, corresponding to the bending out-of-plane vibrations of the C-H bonds of phthalocyanine’s α-form [48,49,50]. Finally, Figure 3c shows the signals corresponding to the spectrum of the composite ZnTTBPc–PMMA film for phthalocyanine, as well as the polymer. It is worth noting that this spectrum has flat bands in the regions corresponding to 2950 cm^−1^, 1727 cm^−1^, and the 1500–960 cm^−1^ interval, resembling the results obtained by S.B. Aziz et al. [46] for PMMA doped with carbon nano-dots. It is important to mention that the results obtained from IR spectroscopy suggest that the films’ components did not undergo degradation due to the deposition techniques used for their fabrication.

**Figure 4:**
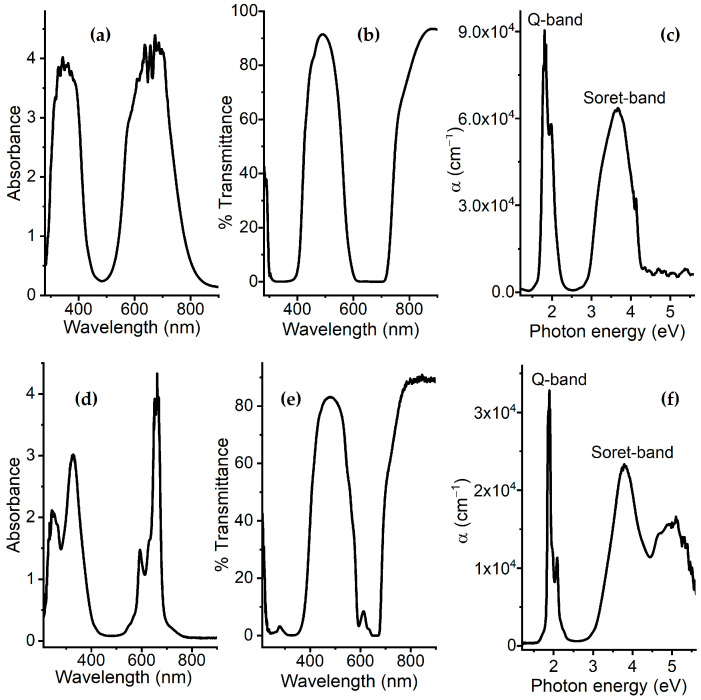
(**a**) Absorbance, (**b**) transmittance, and (**c**) absorption coefficient of ZnTTBPc film. (**d**) Absorbance, (**e**) transmittance, and (**f**) absorption coefficient of ZnTTBPc–PMMA film.

In certain types of WDs, it is required that the active layer has optical properties such that under service conditions, the layer may interact with light to perform functions related to information detection, communication, or visualization. For example, some WDs may communicate with other devices by optical signals in the visible or infrared portions of the spectrum. Thus, active layers must filter some wavelengths to reduce interference or allow for efficient signal transmission. In other WDs involving photovoltaic technology, solar cells may be included for autonomous energy generation, so the active layers must efficiently absorb light to convert it into electricity. Regarding absorption, Figure 4a shows the absorbance spectrum for the pristine ZnTTBPc film. The spectrum shows two phthalocyanine bands. The first one, the Q band, is in the infrared region between 540 and 770 nm, while the second one, the Soret or B band, is in the high-energy region between 290 and 430 nm [48,49]. The Q band is produced by the π-π* transitions from the HOMO (Highest Occupied Molecular Orbital, π) to the LUMO (Lowest Unoccupied Molecular Orbital, π*) and, specifically, transitions from the a_1u_ (HOMO) orbital to the e_g_ (LUMO) orbital [48]. The Soret band is due to π-π* electronic transitions from the a_2u_ and b_2u_ orbitals to the e_g_ (LUMO) orbital. ZnTTBPc does not exhibit a metal-to-ligand and/or ligand-to-metal transition due to its Zn^+2^ d^10^ electronic configuration being completely full [48].

Regarding the transmittance shown in the spectrum in Figure 4b, a maximum of 92% is shown at 488 nm. This film is sensitive to radiation of this wavelength, located in the blue portion of the electromagnetic spectrum. When comparing the absorbance and transmittance spectra of the ZnTTBPc film on glass with those obtained for the ZnTTBPc–PMMA composite film on the same substrate (Figure 4d,e), it is found that the presence of the polymer influences the optical behavior of the composite films. The polymer reduces the maximum value of transmittance to 83% and slightly shifts it to 475 nm. However, a larger effect is observed regarding absorbance, since a new band appears at 245 nm, corresponding to an n-π* electronic transition induced by the nonbonding electrons in PMMA [46]. Additionally, absorbance decreases in the region between 225 and 375 nm, which includes the Soret band of the phthalocyanine and is related to the π-π* transition of the PMMA carbonyl groups [46,51]. The presence of the polymer also promotes Davydov splitting, which is attributed to excitonic coupling between two non-equivalent molecules within a unit cell leading to two bands: Q1 at 590 nm and Q2 at 663 nm. Based on the Davydov model, a blue shift of the Q band occurs when a co-facial alignment occurs between two or more than two molecules, while a red shift of the Q band is observed in the case of coplanar dimers when there is a coupling between electronic states [48]. The types of electronic transitions that can occur inside the ZnTTBPc and ZnTTBPc–PMMA films can be derived from the examination of the absorption spectra. Electron transitions are associated with absorption edges, which are fundamental absorption discontinuities that are indicative of the optical band gap energy. The absorption process is described by an absorption coefficient representing the relative rate at which the incident light intensity declines in a unit length of the medium [52,53].

The absorption coefficient α is obtained from the relationship between transmittance T and film thickness d (with d equal to 1200 nm and 3000 nm for ZnTTBPc and ZnTTBPc–PMMA, respectively), which is defined by the Beer–Lambert law as given by α = ln(T)/d. The relationships between α and the photon’s energy hν for the pristine ZnTTBPc and composite ZnTTBPc–PMMA films are presented in Figure 4c,f. For hν > 4.2 eV, there are differences between the two graphs due to the presence of PMMA; however, α ≥ 10^3^ cm^−1^ in both cases, which is related to direct inter-band transitions [54]. Moreover, the high-intensity absorption of the Soret band can be found from the conjugated phthalocyanine macrocycle at 3.65 and 3.79 eV for ZnTTBPc and ZnTTBPc–PMMA, respectively. The absorption peaks corresponding to the Q band are observed at 1.8 and 1.97 eV for the ZnTTBPc film and at 1.9 and 2.1 eV for the ZnTTBPc–PMMA film. As mentioned before, the Soret and Q bands are related to the π-π* excitations between bonding and antibonding molecular orbitals [54]. For the ZnTTBPc–PMMA films, different bands appear in the UV region at 4.6 and 5.1 eV and are related to pure PMMA absorption [46].

One of the disadvantages of embedding organic semiconductors such as phthalocyanines in polymeric matrices is the decrease in charge transport due to the presence of new phases and interphases inside the films, where charge transport occurs. It seems that there is no significant decrease in the semiconducting capacity of the ZnTTBPc–PMMA film; on the other hand, band gap analysis can provide useful information about the efficiency of the films as organic semiconductors. The Tauc equation, in Equation (1), was thus applied to assess the optical band gap of pristine ZnTTBPc and the related composite film from the α spectra:

(αhν) = B(hν − E_g_)^γ^ (1)

**Figure 5:**
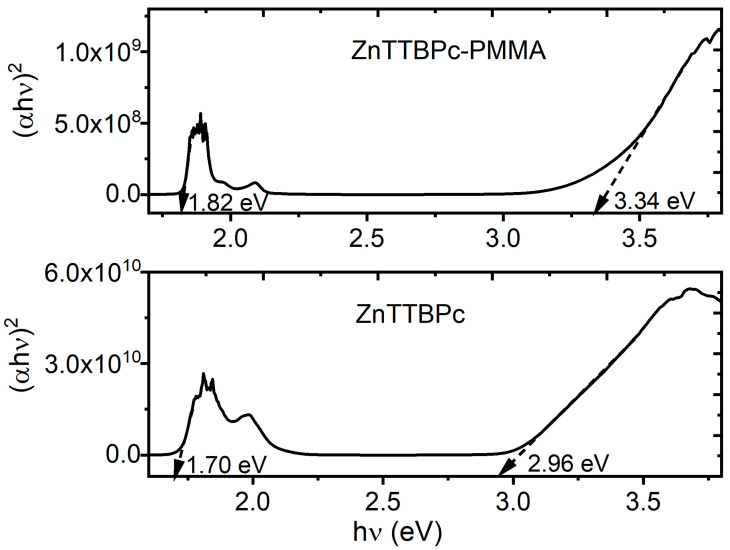
Plot of (αhν)^2^ versus hν of ZnTTBPc and ZnTTBPc–PMMA films.

In this equation, the parameter dependent on the inter-band transition probability is denoted by B, the optical band gap is denoted by E_g_, and the index γ defines the nature of the electronic transition responsible for optical absorption [55]. As α ≥ 10^3^ cm^−1^ in these films, their electronic transitions are considered direct, so that γ = ½ [56]. Direct electron transitions involve the interaction of the photon hν with an electron in the valence band, which allows the electron to transition to the conduction band without significant momentum transfer. In this case, the intercept of the extrapolated linear part of the plot of (αhν)^2^ versus hν with abscissa, in Figure 5, allows for the determination of the E_g_ value [57]. The energy band gaps thus obtained are 1.70 eV (ZnTTBPc film) and 1.82 eV (ZnTTBPc–PMMA film) for the onset of the absorption spectrum and 2.96 eV (ZnTTBPc film) and 3.34 eV (ZnTTBPc–PMMA film) for the fundamental energy gap. The presence of the polymeric matrix leads to a slight increase in the band gap, probably due to an increase in the number of film defects, phases, and interphases; however, the obtained band gap has a value within the same order of magnitude as the one reported for composite films with PMMA matrices and carbon nano-dots [46], CrCl_3_ and CoCl_2_ [58], or CaCO_3_ [52] reinforcements, so ZnTTBPc–PMMA film may still be used in the fabrication of WDs. These results are also important because the presence of the polymer as a matrix facilitates the incorporation of ZnTTBPc–PMMA film in different types of conventional substrates, such as PET, glass, and n-type silicon, as well as in innovative substrates, such as the palm leaves and polyester fabrics considered in this work.

### 3.2. Study of ZnTTBPc–PMMA Films on Different Substrates

The reasons to study different substrates are related to the WD service conditions and the properties provided by each substrate. For instance, PET is light and flexible and has excellent mechanical properties; it can also adapt to the shape of the body or remain in continuous contact with it. This also happens for the polyester fabric, which also enables perspiration, which is important in WDs that are used during physical activities. Glass, like PET, is transparent and has excellent mechanical properties, and it is also adequate for use under extreme abrasion conditions or high temperatures due to its hardness and thermal resistance. As a semiconductor, n-type silicon is also a rigid substrate that, through miniaturization, allows for the fabrication of integrated circuits, compact sensors, and information processors. Finally, palm leaves provide a flexible, biodegradable, and environmentally friendly material.

**Figure 6:**
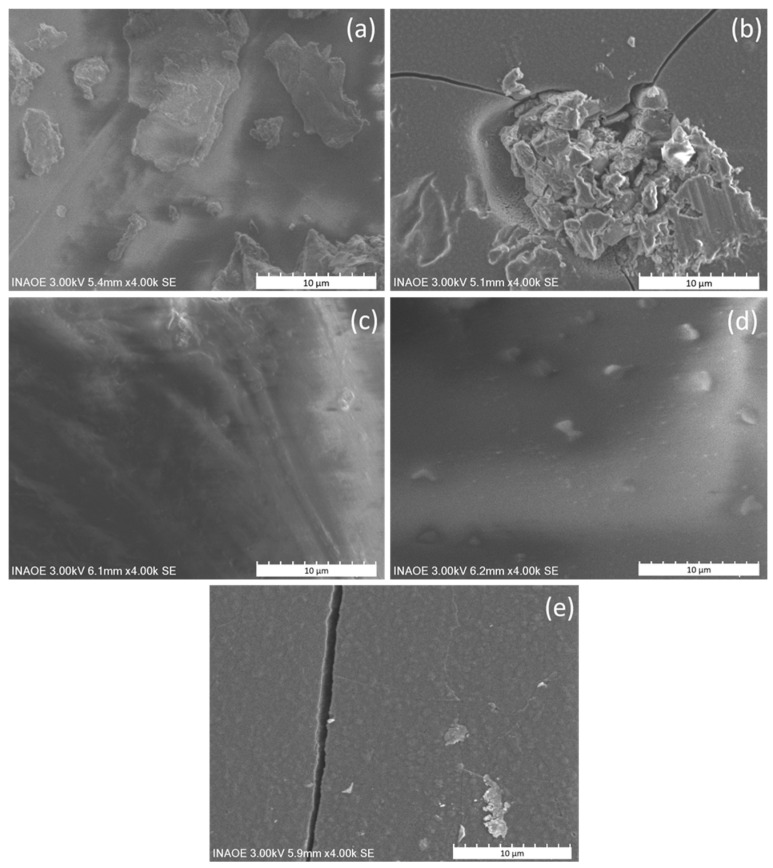
SEM images of ZnTTBPc–PMMA films on substrates of (**a**) palm, (**b**) glass, (**c**) polyester, (**d**) silicon, and (**e**) PET.

To verify the effect of the substrate on the morphology of the ZnTTBPc–PMMA films, SEM analysis was conducted; the 4000× images for each substrate are shown in Figure 5. The film on palm (Figure 6a) exhibits a rough and irregular texture with dispersed particles of various sizes. Although distinct crack patterns are not clearly visible, the presence of uneven aggregates and fragmented domains suggests poor cohesion and limited film continuity, likely due to the fibrous and porous structure of the palm substrate. The film on glass (Figure 6b) displays a well-defined network of sharp-edged cracks, accompanied by central accumulations and irregular agglomerates. These features are consistent with the development of internal stress during drying, likely caused by strong film–substrate interactions and thermal expansion mismatches between the polymer matrix and the rigid glass. In the case of the polyester fabric (Figure 6c), the film presents an undulating topography that follows the woven texture of the substrate. The coating conforms to the surface but fails to uniformly cover the inter-fiber spaces, resulting in a heterogeneous morphology with alternating smooth and irregular regions. The film on silicon (Figure 6d) appears smooth, continuous, and free of visible cracking, with small, evenly distributed globular particles. However, this film detached from the substrate after deposition, indicating limited interfacial adhesion. This detachment may be attributed to the low surface energy and relatively hydrophobic character of untreated crystalline silicon, which lacks polar functional groups capable of forming effective interactions with the PMMA matrix. In contrast, substrates like glass and PET provide better chemical compatibility, enhancing film anchoring through intermolecular or interfacial bonding. The uniformity of the free-standing silicon film supports the hypothesis that silicon acted primarily as a passive physical support, allowing for stress-free film formation but without promoting chemical or mechanical adhesion. Finally, the film on PET (Figure 6e) shows a prominent, well-defined longitudinal crack, along with localized surface agglomerates. These morphological features likely result from internal stresses accumulated during solvent evaporation or from thermal expansion mismatch between the PET substrate and the ZnTTBPc–PMMA composite film.

While direct contact angle measurements were not performed, the following conclusions regarding wettability and surface energy can be drawn from the observed morphological behavior: On silicon, the complete detachment of the film indicates poor wettability and low surface energy, consistent with the hydrophobic nature of untreated crystalline silicon. On glass, strong film adhesion combined with extensive cracking suggests good wetting and high surface energy but also stress accumulation during drying. PET exhibits a similar behavior, with stable adhesion and well-defined cracking, indicating moderate wettability and surface compatibility. In contrast, the polyester fabric substrate shows incomplete film coverage, reflecting heterogeneous wetting influenced by its fibrous microstructure. The palm leaf substrate supports film retention in fragmented regions, suggesting partial wetting, although its highly porous and irregular surface prevents meaningful contact angle analysis. These consistent substrate-dependent outcomes, supported by SEM imaging and mechanical behavior, provide reliable qualitative insight into interfacial interactions. Future work will explore the quantitative evaluation of surface energy and contact angles, particularly on smoother substrates such as PET, which is especially relevant for wearable device integration. In contrast, palm leaf—despite being of interest for sustainable applications—does not allow for reliable contact angle analysis due to its highly irregular and porous surface.

**Figure 7:**
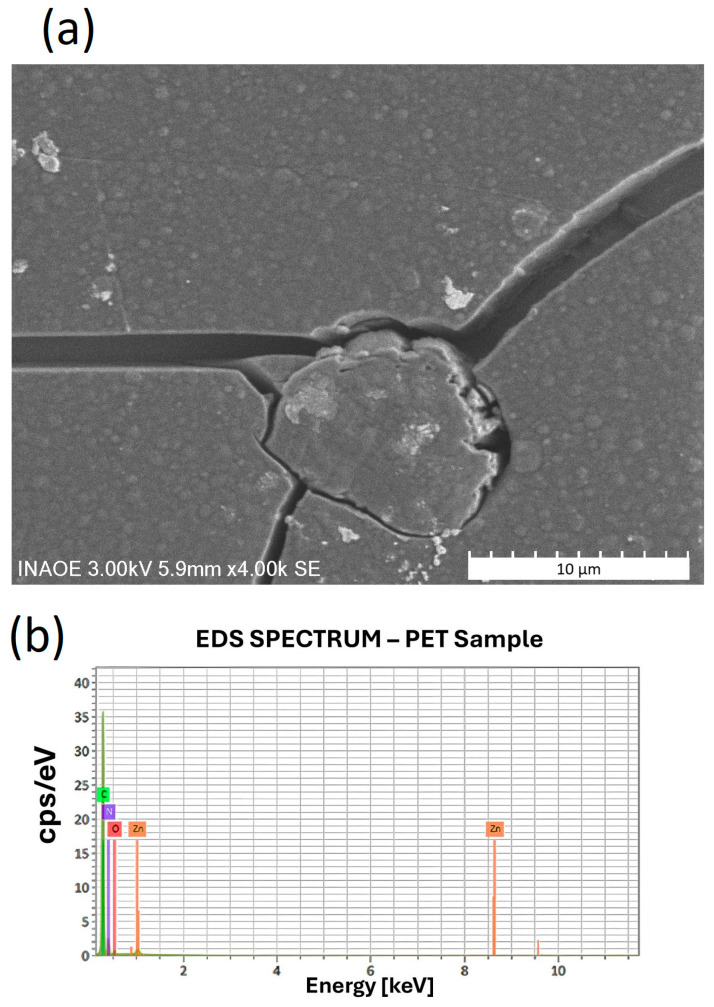
SEM image (**a**) and EDS spectrum (**b**) of ZnTTBPc–PMMA film on PET. Surface cracks and aggregates likely exposed ZnTTBPc, enabling Zn detection.

Figure 7 shows the SEM micrograph (left) and the corresponding EDS spectrum (right) of the ZnTTBPc–PMMA film deposited on PET. EDS was performed on all samples; however, only the PET substrate exhibited detectable levels of zinc, with an estimated content of 7.2–7.3 wt%. This selective detection may be attributed to the agglomeration of ZnTTBPc on the surface and its exposure through the cracking observed in the PMMA film on the PET substrate (see Figure 7, SEM image). The surface agglomeration of ZnTTBPc observed on PET may result from an accelerated drying process, which induces rapid solvent evaporation and thermal contraction within the PMMA matrix. These conditions are likely to promote phase segregation, causing ZnTTBPc molecules to migrate toward the surface before full polymer solidification occurs. Concurrently, the resulting mechanical stresses may exceed the cohesive strength of the matrix, leading to the formation of surface cracks. The coexistence of these two phenomena—the surface localization of ZnTTBPc and the development of microfractures—facilitates the exposure of the active compound at or near the surface. This morphological arrangement enhances the interaction with the electron beam during EDS analysis, thereby improving the likelihood of elemental zinc detection. In contrast, on substrates where the active compound remained embedded deeper within the PMMA bulk, the zinc content may have fallen below the detection threshold of the EDS system.

**Figure 8:**
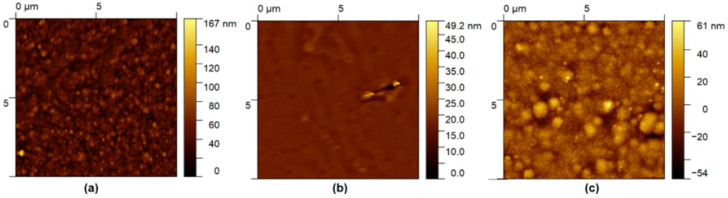
AFM images of ZnTTBPc–PMMA films deposited on (**a**) glass, (**b**) silicon, and (**c**) PET.

Figure 8 shows the AFM images of ZnTTBPc–PMMA films deposited on glass, silicon, and PET substrates, highlighting differences in surface roughness and morphological features resulting from the interaction between the polymer composite and each substrate. The palm leaf and fabric substrates exhibited pronounced surface irregularities and fibrous microstructures that prevented stable tip–surface interactions during AFM scanning. As a result, reliable topographic data could not be acquired for these samples. The ZnTTBPc–PMMA film on glass (Figure 8a) exhibited a root mean square (RMS) roughness of 12.27 nm, while the PET sample (Figure 8c) presented a slightly lower RMS value of 10.37 nm. In contrast, the film on silicon (Figure 8b), displayed a significantly smoother surface, with an RMS roughness of 1.227 nm. This result is consistent with the polished nature of the silicon wafer used as substrate, which provides an atomically flat and defect-free deposition surface. The higher roughness observed in the PET and glass films is indicative of more pronounced surface features, possibly resulting from differences in film nucleation, drying dynamics, or polymer–substrate interactions. In addition to differences in RMS values, the topographic features of the three films revealed rather distinct morphologies. The films on glass and PET consisted of rounded grain-like structures, but their distribution and size differed significantly. The glass substrate promoted the formation of smaller, more uniformly distributed grains, whereas the PET substrate yielded larger, more irregularly sized features, suggesting a less homogeneous film growth process. The film on silicon, aside from being overall smoother, showed a relatively flat surface with sparse, finely distributed features, which may be associated with minimal film stress and higher coating uniformity.

The complementary use of SEM and AFM techniques provided consistent insights into the morphological behavior of ZnTTBPc–PMMA films on various substrates. SEM revealed large-scale features, such as cracking, particle agglomeration, and film detachment, while AFM enabled the nanoscale quantification of surface roughness and grain distribution. Notably, the silicon substrate, with a more homogeneous and continuous topography, exhibited lower roughness and higher morphological uniformity, in contrast with PET and glass, which showed evidence of drying-induced stress and heterogeneity in grain formation. These nanoscale findings correlate with the smooth and continuous morphology observed by SEM. The observations highlight the critical role of substrate–film interactions and deposition dynamics in determining the structural integrity and surface characteristics of composite films, which, in turn, influence such functional properties as optical absorption, fluorescence, and elemental detectability by EDS.

**Figure 9:**
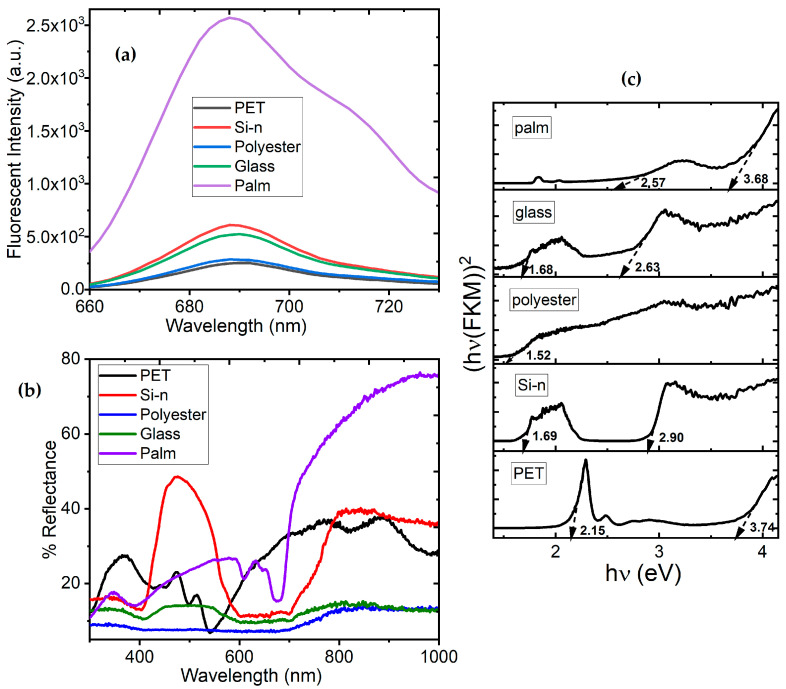
(**a**) Fluorescence spectra, (**b**) reflectance spectra, and (**c**) Kubelka–Munk functions of ZnTTBPc–PMMA films.

Fluorescence characteristics in WDs can be used for different functions, such as warning signals for notifications or increasing user’s visibility under poor lighting conditions. For this reason, we evaluated the fluorescence of the ZnTTBPc–PMMA films over PET, polyester fabric, glass, n-type silicon, and palm leaves. The results of the fluorescence tests obtained at excitation wavelengths of 352 nm are shown in Figure 9a for all the substrates. As has been reported in the literature for ZnPc and its derivatives, the fluorescence band is located between 660 and 730 nm for all the films [59]. Fluorescence intensity is the main difference among the spectra of the different films; their maximum values are reported in Table 1 and were obtained at a wavelength of 688 nm, corresponding to the red portion of the visible spectrum. It is worth noting that the ZnTTBPc–PMMA film deposited over the palm-leaf substrate has a fluorescence intensity several times larger than the rest of the films, whereas the lowest intensity corresponds to the film with the PET substrate. Fluorescence is also related to the monomeric ZnTTBPc inside the film [59,60]. It is important to mention that palm leaves, being an organic material of biological origin, may have chlorophyll-mediated properties that affect energy loss or light absorption, allowing for greater fluorescence [61]. Furthermore, the interaction between the ZnTTBPc–PMMA film and the substrate can influence emission efficiency, and in the case of the palm leaves, this interaction may be conducive to greater fluorescence intensity.

Another important optical characteristic to consider for WDs is the diffuse reflectance R, for which the corresponding spectra are shown in Figure 9b, clearly showing the important effect of the substrate on the films. It is noticed that the film over PET has an R value typically smaller than 30%, whereas the films over polyester fabric and glass have quite smaller R values. In the case of the film deposited over n-type Si, there are two regions of relatively high R; the first one is located at wavelengths of between 400 and 600 nm, and the second one is located above 700 nm. However, the highest R value remains below 50%. Finally, the film over palm leaf has the largest R, also at wavelengths above 700 nm. Table 1 shows the maximum values of R, which, except for the film deposited on n-type Si, are located at wavelengths larger than 800 nm. A low R value helps to reduce interference from environmental light, improving the precision of measurements such as heart rate and oxygen saturation. On the other hand, the film deposited on palm leaf, with a higher R value, could be used in screen-based WDs, where good contrast and high illumination can be achieved for outdoor conditions.

Since the films deposited on fabric, palm leaf, and n-type silicon are opaque, it is preferable to determine the band gap according to the Kubelka–Munk function F(K-M). The values for F(K-M) were obtained from the R spectrum, and the value of the band gap was estimated from Equation (1), as follows, with Equation (2) [62]:

hνα = B(hν − E_KM_)^γ^ (2)

where E_KM_ is the band gap and α is directly proportional to F(KM). In the Tauc equation, α can be replaced by the function in Equation (3):

(hν × F(KM)) = B’(hν − E_KM_)^γ^ (3)

B’ is another proportionality constant. In Figure 9c, (hν × F(KM))^2^ is plotted as a function of the incident energy hν, and the straight segments are extrapolated towards the horizontal axis to determine the band gap; the results are recorded in Table 1 and Figure 9c. The onset gap is located between 1.52 and 2.57 eV and the fundamental band gap between 2.63 and 3.74 eV, depending on the substrate. The film over polyester fabric only shows an onset gap, and its semiconducting capability decreases significantly with respect to the other films. These results show the important effect of the substrate on the ZnTTBPc–PMMA film, although the values thus obtained are within the same order of magnitude as for the pristine ZnTTBPc film (see Figure 5).

Given the remarkable differences among the composite thin films considered in this work, it stands out that each may be applied in a different type of wearable device. The significant fluorescence of the palm-based film suggests possible applications in wearable devices capable of detecting the presence of UV radiation, as may be required in some industrial occupations. On the other hand, the smoothness of the film on silicon suggests possible applications based on interfacing with molecular electronics or biosensor components, both in WDs and other systems. The possibility of growing the films on polyester or PET suggest the possibility of direct, seamless integration of the systems into the fabrics making up WDs. Finally, the composite films over glass substrates suggest potential applications in tactile displays embedded in WDs.

Biocompatibility is an issue that needs consideration in case these films were to be used in implants or other applications involving contact with biological (i.e., human) tissues. Palm leaves are certainly biocompatible, as are glass, polyester, and PET, for many applications, while in some cases, their biocompatibility can be improved with adequate surface treatment. On the other hand, silicon has limited biocompatibility, which suggests its use would involve some physical separation from human tissues for WD applications.

## 4. Conclusions

This work demonstrates the strong substrate dependence on the morphological, optical, and fluorescent properties of ZnTTBPc–PMMA composite films. The films were deposited on conventional, flexible, and sustainable substrates, including glass, PET, n-type silicon, polyester fabric, and palm leaf, revealing significant differences in film continuity, surface roughness, optical response, and material exposure. SEM and AFM analyses showed that silicon provided the smoothest, most homogeneous film due to minimal interaction with the polymer, acting primarily as a physical support. In contrast, glass and PET showed strong film–substrate interactions that promoted stress accumulation and crack formation. Palm and polyester substrates led to irregular and less cohesive morphologies, reflecting the challenge of uniform deposition on porous or fibrous surfaces. Optical characterization revealed the substrate-dependent modulation of absorbance, reflectance, and band gap, with shifts in the Q and Soret bands and variations in Davydov splitting. Fluorescence results also varied with the substrate, with palm leaf enabling the highest emission intensity and PET the lowest, likely due to surface exposure, aggregation, or substrate-induced quenching effects. Additionally, EDS analysis confirmed the selective surface detection of Zn only on PET, attributed to agglomeration and the crack-induced exposure of ZnTTBPc, further supporting the morphological findings. Overall, the study confirms that substrate properties—including surface chemistry, mechanical behavior, and topography—play a critical role in determining the final structure and functionality of ZnTTBPc–PMMA films. These findings provide valuable insights for optimizing organic semiconductor film fabrication in wearable and flexible device applications, where substrate compatibility is essential to performance and reliability.

## Figures and Tables

**Table 1:** Maximum fluorescence intensity of ZnTTBPc–PMMA films over different substrates.

Substrate	Maximum Fluorescence Intensity	Maximum Reflectance (%)	E_K-M_ (eV)
PET	253	38% at 887 nm	2.15, 3.74
Polyester	290	13% at 840 nm	1.52
Glass	521	15% at 809 nm	1.68, 2.63
Si-n	612	48% at 472 nm	1.69, 2.90
Palm	2573	75% at 960 nm	2.57, 3.68

## Data Availability

Data are contained within the article.
